# Aquaporin-4 Autoantibody Detection by ELISA: A Retrospective Characterization of a Commonly Used Assay

**DOI:** 10.1155/2021/8692328

**Published:** 2021-09-28

**Authors:** Jon P. Williams, Justin R. Abbatemarco, Jonathan J. Galli, Stefanie J. Rodenbeck, Lisa K. Peterson, Thomas R. Haven, Meagan Street, John W. Rose, John E. Greenlee, M. Mateo Paz Soldan, Stacey L. Clardy

**Affiliations:** ^1^Department of Neurology, University of Utah School of Medicine, Salt Lake City, UT, USA; ^2^George E. Whalen Department of Veterans Affairs Medical Center, Salt Lake City, UT, USA; ^3^US Air Force, Wright-Patterson AFB, OH, USA; ^4^ARUP Institute for Clinical and Experimental Pathology, Salt Lake City, UT, USA; ^5^Department of Pathology, University of Utah, Salt Lake City, UT, USA

## Abstract

**Objective:**

Aquaporin-4 (AQP4) serum autoantibodies are detected by a variety of methods. The highest sensitivity is achieved with cell-based assays, but the enzyme-linked immunosorbent assay (ELISA) is still commonly utilized by clinicians worldwide.

**Methods:**

We performed a retrospective review to identify all patients at the University of Utah who had AQP4 ELISA testing at ARUP Laboratories from 2010 to 2017. We then reviewed their diagnostic evaluation and final diagnosis based on the ELISA titer result.

**Results:**

A total of 750 tests for the AQP4 ELISA were analyzed, and 47 unique patients with positive titers were identified. Less than half of these patients (49%) met the clinical criteria for neuromyelitis optica spectrum disorder (NMOSD). In cases of low positive titers (3.0–7.9 U/mL, *n* = 19), the most common final diagnosis was multiple sclerosis (52.6%). In the moderate positive cohort (8.0–79.9 U/mL, *n* = 14), only a little more than half the cohort (64.3%) had NMOSD. In cases with high positives (80–160 U/mL, *n* = 14), 100% of patients met clinical criteria for NMOSD.

**Conclusions:**

Our data illustrates diagnostic uncertainty associated with the AQP4 ELISA, an assay that is still commonly ordered by clinicians despite the availability of more sensitive and specific tests to detect AQP4 autoantibodies in patients suspected of having NMOSD. In particular, low positive titer AQP4 ELISA results are particularly nonspecific for the diagnosis of NMOSD. The importance of accessibility to both sensitive and specific AQP4 testing cannot be overemphasized in clinical practice.

## 1. Introduction

Neuromyelitis optica spectrum disorder (NMOSD) is an autoimmune inflammatory condition of the central nervous system (CNS) that preferentially affects the optic nerves, the spinal cord, and the juxtaventricular regions of the diencephalon and brainstem [1]. The discovery in 2004 of the pathogenic autoantibody aquaporin-4 immunoglobulin G (AQP4) [2] allowed for the mechanistic distinction from multiple sclerosis (MS) [3–5]. Recent international consensus guidelines outline major and minor criteria for diagnosis of NMOSD, of which antibody detection remains a central tenet [6]. The methodology of antibody detection has evolved greatly since its initial discovery. Published reports of the general utility of tests for AQP4 include descriptions of immunohistochemistry, immunofluorescence (IF) [7], and immunoprecipitation assays (IPA), as well as ELISA [8, 9] and, most recently, cell-based assays (CBA) and flow cytometry-/fluorescence-activated cell sorting (FACS) [10–12].

Numerous studies have illustrated the superiority of AQP4-transfected cell-based assays, yet the FDA-approved ELISA testing method is still commonly ordered by practicing clinicians [13, 14]. For example, over a recent 12-month period (2/2020-2/2021), the ELISA assay accounted for 62% of all AQP4 assays ordered at ARUP Laboratories (Salt Lake City, UT). In conjunction with ARUP Laboratories, we performed a retrospective review of all cases of NMOSD in our tertiary hospital to determine the utility of ELISA testing in a modern cohort. We anticipated that low positive results on ELISA would be inaccurate in a significant subset of patients and would likely be associated with immune dysfunction and/or neuroinflammatory disorders clinically distinct from NMOSD.

## 2. Methods

This study was approved by the University of Utah IRB (IRB 00108537) along with approval from the ethical standards committee to conduct this study.

**Figure 1:**
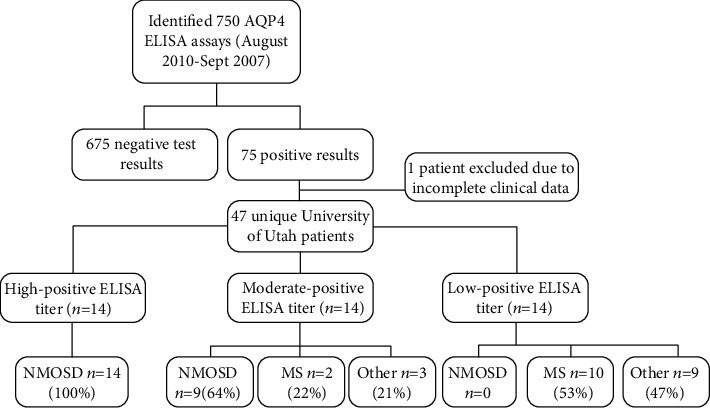
Evaluation of results of AQP4 testing by ELISA from the study period at our institution, the University of Utah. MS: multiple sclerosis; NMOSD: neuromyelitis optica spectrum disorders.

All AQP4 ELISA assay orders placed at ARUP Laboratories for University of Utah patients were identified (test code 2003036 for semiquantitative ELISA and 2013327 for semiquantitative ELISA with reflexive indirect fluorescent antibody). Each case was crosschecked by the medical record number and birth date to avoid duplication of patient records. For subjects with at least one positive AQP4 ELISA result, the electronic medical record (EMR) was retrospectively reviewed by JPW. Patients with a negative ELISA test result or those with missing data were excluded from further analysis. Information on those excluded patients was not available. The study includes all subjects (both inpatient and outpatient) from August 2010 through September 2017 (Figure 1).

Diagnoses were made according to the most updated diagnostic criteria as follows: NMOSD (based on the 2015 Diagnostic Criteria) [6], definite/possible myelitis [15], MS [16], and nervous system Lyme disease [17]. Clinical information including the clinical phenotype, antibody titer, and documented alternative testing methodologies was identified and recorded. When available, coexistent autoimmunity or neurological disease was also documented, as were imaging characteristics and cerebrospinal fluid parameters. Of note, laboratory values for myelin oligodendrocyte (MOG) immunoglobulin G autoantibodies were not included in this analysis as commercial testing was not available during the entire search period.

### 2.1. ARUP Laboratories Aquaporin-4 ELISA Assay

The AQP4 ELISA assay (Kronus, Star, ID, USA) used the M1-AQP4 isoform until April 2015. After that time, ARUP Laboratories utilized the M23-AQP4 ELISA isoform. We were unable to include a comparison of these isoform assays given the limited number of patients enrolled after the M23 assay was implemented in practice.

The ELISA results from both isoforms were reported in arbitrary units (positive values 5 U/mL or greater, per manufacturer) [18, 19]. Positive test results on the AQP4 ELISA were categorized as either low, moderate, or high positives. The low positive designation included any test result between 3.0 and 7.9 U/mL (correlating to a maximum of approximately 5% of the numerically defined maximum readout of 160 U/mL). A moderate positive result was defined as a test result between 8.0 and 79.9 U/mL (over 5% and up to 50% of the maximum), and a high positive result required a value between 80.0 U/mL and the upper limit of the readout, which is reported as greater than 160 U/mL. The reference range for the AQP4 ELISA at ARUP Laboratories evolved during the study period: the reference range was considered positive for any result greater than 5.0 U/mL, but that limit was decreased to 3.0 U/mL in October 2016. By defining “low positive” as under 7.9 U/mL in our study, we prevented this change in the reporting status from impacting the assignment of any patients with test results between 3.0 and 5.0 U/mL.

### 2.2. Aquaporin-4-Transfected Cell-Based Assays

The ARUP Laboratories utilize the Euroimmun M1-CBA (Euroimmun, Lubeck, Germany). A fourfold dilution (1 : 10, 40, 160, and 640) was used to create endpoint titers. Preliminary (unpublished ARUP Laboratories data) data compared the Euroimmun M1-CBA and M23-CBA. The M1-CBA was chosen due to the higher background in the M23-CBA assay, which hindered the interpretation of assay results [19].

The FACS assay performed at Mayo Medical Laboratories (Rochester, MN) has already been described in the literature [19]. Of note, given the retrospective study design with a focus on the real-world clinical assay performance, we did not perform subsequent confirmatory testing on any patient samples and used only information available within the EMR.

### 2.3. Statistical Analysis

Patient demographics and clinical and radiographic features were summarized with descriptive statistics. Qualitative variables were shown as absolute frequencies and percentages. Continuous variables were shown with mean and standard deviation. Two-tailed paired Student's *t*-test with unequal variance was employed for the comparison of mean result values in these groups, and statistical significance was assigned at a *p* value less than 0.05.

## 3. Results

### 3.1. Demographics

A total of 750 tests were ordered during the study period, of which 75 were reported as a positive result, corresponding to 47 unique patients within the University of Utah system. Of these 47 patients, 25 (53%) did not meet the most recent international consensus clinical criteria for NMOSD (Figure 1) [6].

### 3.2. Stratification by ELISA AQP4 Testing

Table 1 shows the stratification of our cohort based on their ELISA AQP4 test results. In the low positive cases (*n* = 19), the most common diagnosis was MS (*n* = 10, 52.6%), followed by optic neuritis or myelitis (without a longitudinal lesion). Of note, neither of the optic neuritis cases had clinical features typical for NMOSD based on criteria outlined in the 2015 NMOSD Diagnostic Criteria [6] as they were unilateral, short-segment optic nerve lesions with mild vision impairment (<20/200).

**Figure 2:**
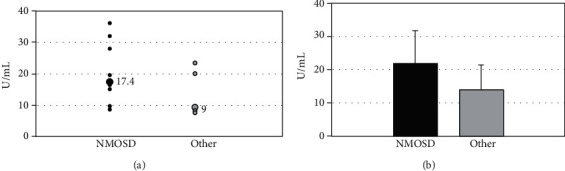
Comparison of values for moderate positive AQP4 ELISA in patients meeting the criteria for NMOSD versus those with other diagnoses. (a) Distribution of values for each group. Patients with multiple tests were averaged to give one test value per patient (3 patients in the NMOSD group). The large labeled data point is the median for the group. (b) Mean assay values for each group. Error bars represent the standard deviation; *p* = 0.21103. NMOSD: neuromyelitis optica spectrum disorders.

In moderate positive cases (*n* = 14), a total of 9 (64.3%) patients had NMOSD. The antibody titers in the moderate positive group did not differ significantly in the patients with NMOSD versus those with other diagnoses (mean 21.8 vs. 13.8 U/mL, respectively, *p* = 0.21; Figure 2). In high positive cases (greater than 80 U/mL, *n* = 14), all the patients (100%) met the clinical criteria for NMOSD.

### 3.3. Ancillary Test Results

Five of the nine confirmed NMOSD patients from the moderate positive ELISA cohort had CSF testing (Table 2). Three patients had a pleocytosis (range 21–106 cells/mm^3^, reference > 5 cells/mL), but all the patients had a normal protein level (27–48 mg/dL, reference > 50 mg/dL). Unique CSF oligoclonal bands (reference ≥ 2 bands) were found in two cases while one patient had matched serum/CSF oligoclonal bands. CSF data was available in three patients without NMOSD in the moderate positive ELISA cohort. CSF-unique bands were found in two patients corresponding to the diagnosis of nervous system Lyme disease and MS. Of 14 patients with NMOSD in the high-positive group, eight had CSF test results. A pleocytosis was noted in four cases (12–282 cells/mm^3^). Oligoclonal bands unique to the CSF were not found in any patients in this group, but matched bands in the CSF/serum were present in 4 of 5 patients.

### 3.4. Comparison to Live Cell-Based Assay

A total of 5 patients with confirmed NMOSD in the moderate positive ELISA results had additional CBA results: 2 were positive by CBA, 1 additional patient was positive by FACS, and 2 were negative by each methodology (Table 3). Of those not meeting the criteria for a diagnosis of NMOSD, all had additional testing and all were negative by FACS (*n* = 4). One of these patients also had CBA at ARUP Laboratories, which was negative as well.

## 4. Discussion

This manuscript describes the clinical utility of the ELISA AQP4 testing in a modern academic cohort of patients. Despite clinical evidence and guidelines recommending CBA AQP4 assays, clinicians *still* routinely utilize the FDA-approved ELISA AQP4 testing kits [6]. Our results further illustrate that low titer ELISA results can confound the diagnostic evaluation, as low titer results are not specific for NMOSD. Even moderate- and high-tier AQP ELISA results can be difficult to interpret suggesting that CBA assays should be the first line of investigation for clinicians if available. Our cohort also supports the observation that NMOSD patients commonly have concomitant markers of autoimmunity, which can add another diagnostic challenge in the setting of equivocal ELISA AQP4 assay results.

Our ELISA stratification showed relatively consistent results which are higher, correlating to more clinically relevant results. There were no confirmed NMOSD cases in the low positive group, and the most common final diagnosis was MS. It is *vital* to clearly differentiate MS from NMOSD, as the prognosis and treatment differ dramatically, especially as there are now three FDA-approved NMOSD therapies [20]. The likelihood of misdiagnosing MS in our study decreased dramatically when AQP4 ELISA results were greater than 8 U/mL. The likelihood of a confirmed NMOSD diagnosis in the moderate positive group was nearly twofold higher than that of an alternative diagnosis (*n* = 9 vs. 5, respectively). Alternative testing methodologies including CBA and FACS AQP4 assays were much more specific for NMOSD cases. There were no documented false positive results, including those with a final diagnosis of MS.

In many clinical settings, the most readily available test for NMOSD remains the ELISA assay, whether due to laboratory vendor contracting (including cost considerations), clinician workflows, or default EMR order sets. If the ELISA assay must be ordered as a primary assay and is negative in patients in whom there is a high clinical suspicion, consideration should be given to pursing evaluation by either CBA or FACS. Likewise, for low and moderate positive results on the ELISA in clinically equivocal cases, consideration should be given to CBA or FACS confirmatory testing for improved specificity.

There is widespread recognition of the association of NMO with other systemic autoimmune diseases, such as systemic lupus erythematosus along with non-organ-specific autoantibodies (i.e., antinuclear antibody) [21, 22]. These coexisting conditions support the hypothesis that NMOSD patients are susceptible to systemic autoimmunity [23]. Our results are consistent with these observations and demonstrate additional markers of autoimmunity/immune dysfunction in patients meeting the criteria for NMOSD (*n* = 5/23, 21.7%).

Several considerations should be kept in mind regarding these data. This study is a single-center, retrospective analysis, and the sample sizes are small. However, this cohort presents a large description of low and moderate positive AQP4 by ELISA. Additionally, this clinically verified evaluation of a relatively rare disease by a major client (University of Utah) for a regional reference laboratory (ARUP Laboratories) implies generalizability of these data. We were unable to fully account for the evolution in the ELISA assay at ARUP. There is emerging literature around the AQP4 M23 isoform ELISA assay which may have improved sensitivity and specificity, but we were unable to include a comparison of AQP4 isoforms given the limited number of patients in our study after the M23 assay was implemented in practice [24]. Our objective was to focus on the specificity of the ELISA AQP4 assay, but we should note the high sensitivity of CBAs and FACS assays which has been shown in a multicenter investigation [13]. The ELISA assay has also been reported to have relatively high sensitivity (~80%) though there is a wide variance in the literature based on the technical aspects of the tests [13, 18].

Our search period predates the widespread availability of the commercial MOG IgG assay, and thus, we did not include MOG autoantibody testing data in this study. Additionally, the diagnostic criteria for NMOSD, along with other disorders, have similarly evolved during this time. We used the latest diagnostic criteria, applied in a retrospective manner.

## 5. Conclusions

This study demonstrates that the use of the AQP4 ELISA in the evaluation of suspected NMOSD has the potential to yield low positive results that are unlikely to be associated with a clinical diagnosis of NMOSD but highly associated with MS. Moderate positive ELISA results were usually associated with NMOSD but could also be seen in various other diagnoses, including MS and Lyme disease. A high positive result of 80 U/mL or higher was invariably consistent with a clinical diagnosis of NMOSD. In the modern era, practicing clinicians continue to frequently order AQP4 ELISA testing despite the availability of more sensitive and specific AQP4 assays. Given this continued laboratory practice, clinicians should interpret the results of less-sensitive AQP4 assays with extra caution, especially in patients not fulfilling the clinical NMOSD criteria.

## Figures and Tables

**Table 1:** Diagnosis by relative level of Aquaporin-4 detection by ELISA.

Diagnosis	*n*, %
Low positive (3.0–7.9 U/mL), *n* = 19	
Multiple sclerosis	10 (52.6)
Optic neuritis	2 (10.5)
Myelitis without LETM	2 (10.5)
Cyclic vomiting syndrome	1 (5.2)
Spinal cord infarct	1 (5.2)
Autoimmune thyroiditis	1 (5.2)
Migraine	1 (5.2)
Insufficient data	1 (5.2)
Moderate positive (8.0–79.9 U/mL), *n* = 14	
NMOSD	9 (64.3)
Multiple sclerosis	2 (14.3)
Disseminated Lyme	1 (7.1)
Myelitis without LETM	1 (7.1)
Migraine	1 (7.1)
High positive (80 U/mL or higher), *n* = 14	
NMOSD	14, (100)

**Table 2:** Ancillary test results.

	NMOSD		Other
	High positive (range)	Moderate positive (range)	
	*n* = 8/14	*n* = 5/9	*n* = 4/5
*CSF testing reported*			
Pleocytosis (cells/mm^3^)	4 (12–282)	4 (0–106)	2 (5–246)
Protein (mg/dL)	4 (41–106)	4 (27–48)	2 (32–122)
Oligoclonal bands, *n*	5	4	3
CSF unique, *n*	2 (1–3)	2 (1–3)	2 (3–4)
Matched, *n*	4	1	1
None	1	1	0
*Autoimmune markers/immune dysfunction*			
SSA/B	2	N/R	N/R
RF	1	N/R	N/R
ANA	1	N/R	N/R
Myasthenia	N/R	1	N/R
Hypogammaglobulinemia	N/R	N/R	1

N/R: not reported. “Matched” refers to identical oligoclonal band detection in both serum and CSF.

**Table 3:** Additional testing in moderate positive by ELISA subgroup.

Diagnosis	Total	Retested	CBA^A,M^		FACS^M^	
			(+)	(−)	(+)	(−)
NMOSD	9	5	2^A,M^	1^M^	1	1
Multiple sclerosis	2	1	0	1^A^	0	1
Disseminated Lyme	1	1	0	1^A^	0	1
Myelitis without LETM	1	1	0	0	0	1
Migraine	1	1	0	0	0	1

CBA: cell-based assay; FACS: fluorescence-activated cell sorting/flow cytometry; (+): positive result; (−): negative result; ^A^performed at ARUP Laboratories; ^M^performed at Mayo Medical Laboratories.

## Data Availability

The corresponding author is in possession of detailed methods and anonymized data of the present study, which can be available upon reasonable request. The data are not publicly available due to privacy or ethical restrictions.
